# “I'm putting a Band-Aid on a bullet hole the only way I know how:” a qualitative study of barriers and facilitators to opioid misuse and recovery in Nevada

**DOI:** 10.1186/s13011-022-00503-0

**Published:** 2022-11-24

**Authors:** Tessa Swigart, Lisa Lee

**Affiliations:** 1 University of Nevada, Reno, School of Public Health, ICF Next, Fairfax Virginia, University of Nevada, 1664 N. Virginia Street, Reno, NV 89557 USA; 2 Roots to Wings Consulting, Reno, NV USA

**Keywords:** Opioid, Opioid misuse, Drug overdose, Nonmedical opioid use, Substance use, People who use drugs, Trauma, Recovery

## Abstract

Nevada, like the rest of the United States, is undergoing substantial challenges with opioid misuse and overdose deaths, further exacerbated by the COVID-19 pandemic. While much of the attention around opioid overdose prevention is centered on treatment and recovery, it is important to understand the factors that influence initiation of use, and the function opioids play in people’s everyday lives. We conducted qualitative semi-structured individual interviews using purposive and snowball sampling among 35 people across Nevada with a current or prior history of illegal opioid or nonmedical opioid use. Our study aimed to understand why people start to use drugs, why they continue to use, what motivates them to continue to use or to seek treatment, and why individuals maintain recovery or return to use. We found five significant themes as perceived by the participants: that trauma is a risk factor for drug misuse; that the function of opioids in everyday life is a source of temporary relief but highly disruptive in the longer term; that recovery is most often a complicated and nonlinear process; that there are many barriers to accessing services that are both logistical and psychosocial; and that compassion, hope, and having a sense of purpose are crucial to the recovery process. The experiences of the study participants portray opioid use as a rational choice to escape the emotional ramifications of trauma. However, due to the physiological dependence and physical risk of opioids, drug policies that criminalize addiction, societal stigma, and the barriers to timely access of harm reduction, treatment, and recovery services, opioid users often become trapped in a distressing and dangerous cycle. Lastly, respondents indicated that hope, value, belonging, and purpose are powerful factors in cultivating intrinsic motivation for making positive changes and fostering resilience in the recovery process. Opioid misuse services should help meet basic needs and incorporate holistic approaches to recovery that acknowledge past trauma and the complexity of the recovery process.

**Supplementary Information:**

The online version contains supplementary material available at 10.1186/s13011-022-00503-0.

## Background

From 2019 to 2020, the United States saw a 30% increase in drug overdose deaths, with a quintupling since 1999 [1]. According to the Centers for Disease Control and Prevention (CDC), there have been three waves of opioid overdose deaths; the first in the 1990s with a rise in prescription opioid overdose deaths, the second starting around 2010 with a rise in heroin overdose deaths, and the third starting around 2013 with a rise in synthetic opioid overdose deaths [1]. The increases in opioid use and nonmedical opioid use (NMOU)—the use of prescription opioids either without a prescription or in a manner not intended by the prescriber—were due in part from a change in pain management in the 1980s alongside aggressive opioid marketing and liberal prescribing practices [2, 3]. While access to prescriptions has decreased, demand for illicit opioids has grown; prescription opioids have declined since 2012, yet overdose deaths have continued to increase from heroin, illicitly manufactured fentanyl, and other strong synthetic analogues [3]. Illicitly manufactured fentanyl was responsible for 70% of all drug overdose deaths in 2020 [4].

Nevada, like the rest of the United States, is facing substantial challenges with opioid misuse (that is, the use of illicit opioids and/or NMOU), further exacerbated by the COVID-19 pandemic [5]. Nevada experienced a 55% increase in drug-related deaths from 2019 to 2020 and a 20% increase in the overdose death rate in 2021 compared to the same period in 2020 [6, 7]. Of these deaths, two-thirds involved opioids and one in three involved fentanyl [7]. In December of 2020, Nevada was placed on a ‘red alert’ status by the National Drug HelpLine for being at increased risk of overdoses. Current available statewide assessments and surveillance data provide prevalence, incidence, and mortality information in order to inform prevention and treatment efforts. The assessments thus far have not integrated research that involves the lived experiences of people who currently misuse or have misused opioids in the past. There are substantial data that suggest a causal relationship between adverse childhood experiences and/or past trauma, and initiation of substance use [8–13]. There is a growing body of evidence underscoring the need for enhanced overdose surveillance and prevention, more options for evidence-based services like the provision of medication for opioid use disorder (MOUD, which consists of three approved medications), and a call to decrease barriers to those and other relevant services [14–18]. Trauma-informed care in behavioral health is also gaining traction in practice [19]. Yet there is a lack of research that gives voice to people who misuse opioids and their experience accessing and participating in services, how they perceive their drug use and recovery, and how those experiences could help shape the interventions and treatment directed at them. Prevention efforts and services that fail to acknowledge and address wounds that can lead to misuse, risk missing the opportunity to aid sustainable healing.

Therefore, the purpose of this study was to speak to people in Nevada who currently misuse opioids, or who had a history of misusing opioids, to 1) better understand barriers and facilitators to accessing services that meet their needs, and 2) understand the contexts that precipitated their initiation to drug use and what factors helped aid or impede their recovery. Our objective was to use this unique insight to better understand how to amend, revise, or develop new initiatives to confront the opioid crisis and to meet the needs as outlined by those with direct lived experience.

## Methodology

### Study design

We conducted qualitative semi-structured individual interviews using purposive and snowball sampling among 35 people with a current or prior history of using illicit opioids or NMOU. Our questions were developed to capture why people start to use drugs, why they continue to use, what motivates them to continue to use or to seek treatment, and why individuals maintain recovery or return to use. We also wanted to explore whether there were some treatments, or aspects of treatments, that had been successful or more successful than others, and if so why. We spoke with people in different stages of opioid misuse and wanted to have as complete an understanding of their experiences as possible, so we used the transtheoretical model as well as the socio-ecological model to develop our semi-structured interview guide [20, 21]. Using these frameworks, we drafted questions under different domains (see guide in Additional file 1) and then had a small group of subject matter experts (those that work in services for opioid misuse and/or with lived experience) give feedback on the relevance, wording, and appropriateness of the questions. Additionally, we were interested in the intrapersonal, interpersonal, environmental, and structural barriers and facilitators to obtaining services and how to overcome these barriers, on an individual basis, on a community level, and more structurally.

### Participant recruitment and selection

For the recruitment of study participants, we worked with two different organizations in Nevada who serve people who use drugs (PWUD) and/or people in recovery. One organization is a harm reduction and syringe services program, and the other is a recovery community organization. Both perform outreach in their local communities and surrounding rural areas and offer support to PWUD and individuals in or seeking recovery. The research contacts who worked with us on recruitment were well positioned to find eligible participants and had established trust in the community. Participants were eligible for the study if they were over 18 years old, had misused opioids in the past, or were currently misusing opioids. Due to time constraints, we only interviewed those who could speak English. We selected participants purposively through the research partners from the partner organizations. Because these partners worked closely with PWUD in their facility and through outreach, they had knowledge of many of the participants’ history with opioid use. Our recruitment partners also performed some snowball sampling with contacts of study participants to find current opioid users who were not currently involved in any services. There were 32 participants recruited purposively and 3 through snowball sampling.

### Data collection and interview procedures

A research contact from the partner organization approached potential participants they knew or suspected were eligible and presented them with a recruitment postcard. If the potential participant’s eligibility was unknown, the contact briefly screened the participant to verify eligibility. Due to COVID-19 safety precautions, all interviews were conducted remotely (either by phone or by videoconferencing) except when there were participants living in an encampment. The contact from the partner organization scheduled a time for the interview and provided a safe private space at the organization and telephone/tablet/computer for the participant to join the interview remotely. The research contact, when scheduling the interview, used an alias for the participant to protect their identity; all interviews were conducted anonymously. For interviews conducted in an encampment, both the interviewee and the facilitator wore masks and kept six feet distance. When the interview began, the facilitator introduced themselves and gave a brief overview of the study, of their background and credentials, advised the respondent that the interview would be recorded, read through the consent information script, and verbally confirmed consent. The participant was given a cash incentive of $50 USD for offering their time and perspective. This amount was determined with advice from our subject matter experts based on interview time. The interviews last 60–90 min, were conducted by one of two female facilitators, one with a doctorate in epidemiology and over 10 years’ experience conducting mixed-methods research (TS), and one DrPH candidate with a masters in anthropology, lived experience in recovery from opioid use disorder, and experience facilitating qualitative research (LL). The interview instrument was only a guide, which we piloted on the first three interviews. Ultimately, we did not feel it necessary to change the guide. We planned to interview between 20–30 participants, based on budgetary and logistical parameters of the study and on sample size estimates to reach saturation [22].

### Analysis

We used MAXQDA 2022 to conduct thematic inductive analysis [23] through an iterative process. Both authors (TS and LL) facilitated the interviews, coded the transcripts, and completed the analysis. Upon completion of the data collection, we met to examine all the interviews and to decide on starting codes for the analysis. Due to time constraints, we both read all interviews but divided them evenly for coding, with one coder per transcript. To refine our initial codes, we each coded the same document and compared coding, and then developed the codebook. We agreed that our understanding of the data would likely evolve throughout the coding process. To truly ensure reliability we had ongoing discussions about the data as we progressed with the coding, meeting regularly to refine and rework the concepts we were identifying and develop the final themes.

### Ethics

This study [number 1691087–3] was reviewed by the University of Nevada, Reno Institutional Review Board (IRB) and given Exempt status on February 18, 2021.

## Results

From February 24^th^ through April 15^th^, 2021, we interviewed a total of 35 participants between the ages of 25–62 years. We interviewed 5 more than initially planned because we were still getting rich data after 30 interviews. Among the respondents, 29 lived in an urban area and 6 in a rural area. There were 16 people who currently used opioids, and 19 people who had used them in the past. Of the participants, 26 were white, 5 were African American, 4 were Native American, and 2 were Hispanic/Latinx (Table 1). We believe that the diversity and size of our sample was sufficient to arrive at the themes we found, of which five were most significant as perceived by the participants: that trauma is a risk factor for drug misuse; that the function of opioids in everyday life is highly disruptive as well as source of temporary relief; that recovery is most often a complicated and nonlinear process; that there are many barriers to accessing services that are both logistical and psychosocial; and that compassion, hope, and having a sense of purpose are crucial to the recovery process. We would like to note that in this section we intentionally provided ample space for the voices of people with lived experience.

**Table 1:** Key demographics

	**Current opioid user **	**Former opioid user**
	*n*=16	*n*=19
	Total *N*=35	
Self-identified gender		
M	10	9
F	6	9
Trans F	1	0
Nonbinary	0	1
Average age	40	37
Race		
White	12	14
African American	2	3
Native American	2	2
Hispanic/Latinx	1	1
Geography		
Urban	15	14
Rural	1	5
Housing status		
Housed	5	16
Hotel/motel	4	2
Encampment	7	1

### Trauma as a risk factor

There was an overarching theme of personal trauma as a risk factor for opioid misuse. Out of all interviews, there were only two participants who said they had a positive childhood experience. However, all but one participant spoke about significant trauma or multiple traumas that preceded their entry into misusing opioids. These traumatic experiences were so painful that often opioids were the only perceived antidote available for temporary relief.

I don't think that I would, you know, would have ever been a drug addict without the violence, without the abandonment… because I've always tried to cancel out what I was feeling. Like I told you I don't do drugs just to feel good. I do drugs so I don't feel anything.

*Male, 50s*, *currently using opioids*

After they took my kids away from me, I relapsed like two months later. I relapsed and started shooting dope again. And then, within two months I was shooting pills. I didn't know I was addicted to them until they weren't there. Like I was just trying to get high and not think about my kids that I just lost.

Female, 30s, formerly used opioids

Oh gosh I was a really good kid. I was an altar boy. I had- I had dreams of becoming a priest. I was really into the Catholic faith and that whole lifestyle you know. The good boy going to school and honoring my mother and father and… I started smoking cigarettes and started smoking pot. And that was it. I was off and running because my old man used to beat the tar out of me. You know, and it was my way to escape and fill the pain holes, if you will. And that's what I did.

Male, 50s, formerly used opioids

There were a few years, where there were several traumas that have occurred, unfortunately, and like I think if I would have experienced, like, maybe two of the five I would have been fine you know, or maybe it was like the last one that tips things over the edge. There's a very clear delineation in my mind between, like, before those things happened and how I interacted with substances versus after those things happened. I was just numbing the pain, or whatever, that's what happened.

Female, 40s, currently using opioids

I liked to play with dolls. I liked to braid hair and to my, to my brothers and their friends that made me a faggot. And so they picked on me and they picked on me and whatever. but then when I was 11, I was molested and it got really real, you know? And then, so I'm like, oh shit, maybe I am, you know what this guy did to me, like made me think maybe I'm gay. And then, so it's not like that's bad, but in my religion, if you're gay, you get kicked out of the family. They just disowned my aunt because she came out of the closet. Yeah. They just disowned her. You never saw her again. And so, like being a kid and having these things done, you say had a pretty traumatic upbringing… Most of it could have happened from childhood. I mean, there's not a single person out there that's a drug addict that didn't have some sort of traumatic thing happen. And just, you know, we just respond to it wrong just once. And it works for us just this one time. And then, so we take that, you know, and we go with it and then that was it.

Male, 40s, formerly used opioids

I went to a trauma-based rehab facility, and where they say that addiction is a symptom of trauma. And that's what they're focused on. Like it was hard. If I wouldn't have been facing seven years of prison for not completing it, I probably would have left. Because they make you, they make you deal with shit you know, they make you process things that you bury down forever and they made me talk about it in detail. With feeling words this time I got raped and that time I got raped and who raped me. And why I think it's my fault. I had to use “I” feeling words when talking about it and read it in front of a group where they all give me feedback, and I don't know it was… I definitely didn't want to do it at the time, but I can look people in the eyes when I talk to them now. And I don't spend every day trying to numb the pain anymore.

Female, 30s, formerly used opioids

We're fucking people that were just like you except we had a fucking… we had a hardship where you didn’t, we had a bad experience where you didn't. So kudos for you but don't fucking don't shit on me because I had fucked up shit happen and I'm putting, I'm putting a Band-Aid on a bullet hole the only way I know how

Female, 30s, currently using opioids

There were also a number of participants who cited intergenerational trauma, intrafamilial drug use, or family and friends as the gateway to their own drug initiation.

I didn't really have a chance not to become a drug addict. Everybody I was raised around was a drug addict. My mom was in prison, so my brothers and I went to live with my dad's friends, family.

Male, 30s, formerly used opioids

My brothers and sisters weren't really like the best role models. Like, they were always either getting drunk or doing drugs or something. So, I mean that's really where I got it from.

Male, 20s, currently using opioids

When I was 13, I started using meth and heroin through my dad's friend because I was playing with his bag of dope but then he said, if you’re going to play with dope you’re going to do it right. Showed me how to hit with a needle and stuff. From there, definitely wasn't the best father of the year but he did teach us how to use drugs properly.

Male, 30s, currently using opioids

My biological dad was a heroin addict. He left before I was born. I never met him. He ended up dying of an overdose when I was 15 months, when he came back to Vegas to try and reconnect with me and get clean, because he did not have services because there was not the intervention that was necessary to make sure that that didn't happen.

Non-binary, 30s, formerly used opioids

My mom was she- she was addicted to meth and she was a bartender, you know, so I went, I changed schools I went to like 13 different elementary schools, 3 middle schools, 4 high schools. My dad was an alcoholic who never- he was always obsessed with my mom but never wanted anything to do with me and my brother. And um so I never really had, you know, any type of father figure I had to learn it all on my own.

Female, 25, currently using opioids

In summary, drug use, including opioid use, was viewed by participants as a viable solution to alleviating the pain of traumatic events. In some cases, past trauma included a familial or intergenerational chaotic relationship to drugs that facilitated their own use.

### The function of opioids in everyday life

Almost every person interviewed (all but one) who currently used drugs said they did not like the function that drugs played in their life. Participants said it was stressful not knowing how they would fund or find the drugs they need to get through the day. Many individuals described the physiological dependence on heroin as being like “claws,” “shackles,” or “hooks” in their lives. Most respondents who currently used drugs indicated how much time and effort they spent trying to afford, acquire, and get the drugs into their bodies, all to stave off the dreaded “dope sick.”

I would say just don't ever, don't ever start. Don't ever be curious to begin with, don't ever, it's just not- not the business, not what it's cracked up to be at all. Certainly not. And it really digs your hooks in, it goes really deep and it's hard to get away from it.

Female, 30s, currently using opioids

You're trapped. I mean I'm, I'm fully shackled to this addiction.

Male, 40s, currently using opioids

Yeah. It has a grip.. It's got a serious grip sometimes.

Female, 30s, formerly used opioids

I trapped myself and don’t know how to get myself out.

Male, 40s, currently using opioids

Many participants also acknowledged that opioids were a form of self-medication that were perceived to numb pain, both physical and emotional. Participants noted the effects of opioids that produced feelings of euphoria, numbness, and as a temporary solution to altering their mental or emotional state. Their responses are illustrative of the allure of opioids that facilitate daily use.

I don't feel any better than when I'm high on the opiates. There's no other feeling like it. It's like, it's like being next to God. Next to godliness. You say cleanliness is next to godliness no its not its opiates. Opiates is next to godliness. That's how I feel, I feel invincible, I feel like there's nothing I can't do at that time.

Male, 40s, currently using opioids

Some people use drugs, uh, because they feel good, the drugs feel good. They make you feel great. But why are you using it just to feel that you can feel great now, and then we can move on, or are we using them because, uh, as a tool, you know, are they one of our tools, or is this our response to the feelings when you start using it as a solution, right? When it starts to solve your problems, then it was, people were like, drugs are bad. Like are they? They seemed to solve all of my problems, you know? … And then when you, when you, when you get so deep into it, when get so far into drugs, solving your problems, you realize that everything around you, there's nothing really consistent around you, your jobs aren't consistent because you're, you're, you know, you, you have a priority sometimes, and it's, it's getting high… And the only consistent thing in your life is dope. Dude. Every time I get dope, like it shows up for me every time I get meth, it does exactly what meth is supposed to do. When I get a bottle of Southern Comfort. This bottle does what it's supposed to do. I can count on this bottle. It's consistent. I can count on this needle. It's consistent. What isn't consistent, is you motherfucker. You know what I mean? And the world around me. And so like, we move towards what makes the most sense. And so if you're like, so we were telling these drug addicts they're wired wrong and all this. And we're like, well, shit no, they're kind of wired right. They're doing exactly- they’re responding to the world. Exactly how they know how.

Male, 40s, formerly used opioids

My kid's dad was really abusive. I was just trying to numb that reality of everything. I figured well you know if I can't feel anything physically or mentally, then you know everything will be okay.

Female, 20s, formerly used opioids

Many participants discussed their awareness of the risks of use, especially within the current landscape of the adulterated illicit market, and many had awareness and knowledge of how to acquire and use naloxone. Even still, a prominent theme was that opioid dependence is all-consuming and leaves little space–physical, emotional, or mental–for alternative activities. Withdrawing from heroin was reported by almost everyone as a nearly unbearable experience and the thought of having to go through it without help kept people from trying. The time spent trying to afford, purchase, and consume opioids to avoid withdrawals did not allow much room, time, or space to think about other options.

Can’t function... can't function without it. For me, my when I, when I start to get sick… It's the most overwhelming panic, unreasonable terrifying panic. That you can possibly imagine. It's not unreasonable. I know it's not for nothing. I know there's no reason for me to be panicking like this, but it just comes wave after wave after wave, most terrifying and unreasonable panic.

Male, 40s, currently using opioids

I have lost jobs. And then, uh, the cost of it, it's crazy. The things I've done to support to that, that… sickness will motivate you to do... I mean, cause tweaking, you know, I would steal stuff from stores and stuff, but you have that sickness motivating you. So I would do way worse things for heroin.

Male, 30s, currently using opioids

It doesn't matter what ultimatum you give them [PWUD]. Drugs are more powerful. You can, you can offer you like, uh, put your love, uh, up and, or your, their kids and they will still choose drugs. And it's not because they want to, it’s because the drug is that powerful.

Male, 30s, currently using opioids

It was my whole life. Looking for drugs, finding drugs, getting money for drugs, doing drugs. And it got to the point it consumed everything.

Female, 30s, used opioids in the past

It's interfering in numerous ways that are to me even hard to talk about. I mean, it interferes with everything, your health, your sex drive, your money, your ability to function. You know, your whole life just seems to go by fast. To come up with the money to, to be able to stay well, and then functional, you know. Male, 50s, currently using opioids

Social exclusion was another experience often mentioned by participants as a factor of their opioid use. Social isolation was often perpetrated by family members and other close contacts who struggled with their loved one’s drug use. This exclusion often was discussed as contributing to feelings of disconnection and internalized stigma that kept a participant trapped in a cycle of ongoing use.

The opposite of addiction isn’t sobriety its connection to other people or connection within your community, and I can assure you that is so true.

Female, 40s, currently using opioids

I was alone. It was a terrible, terrible time. I craved to not be alone. Even now. I'm the only Native American I know that has had an addiction to heroin.

Female, 20s, formerly used opioids

Then my granny like sent me a letter when I was in jail saying that she's not going to support my lifestyle anymore, and she's not going to put money on my books. And that's like, that was a tough blow because she was the only woman left, you know, I burned everybody.

Male, 40s, formerly used opioids

Participants discussed the role of opioids in their daily lives in a variety of ways. Some expressed the value to being able to self-medicate while most participants acknowledged the risks of using and the disruption and complications to daily life that physiological dependence brings.

### Recovery is most often complicated and nonlinear

The participants spoke of a general misperception that recovery is something achievable in a short amount of time with little holistic support. There did not appear to be predictable push factors that compelled individuals to initiate or maintain recovery, and people’s “bottom” –that is, the catalyst that propelled them onto a pathway to recovery–differed greatly from one participant to another (for one respondent it was her first pregnancy, for another it was when her fifth child was taken by Child Protective Services). Participants said that recovery can be a long process, often involving recurrence of use, and should not be expected to be a simple progression in which someone enters a center as a drug user and comes out completely recovered. Further, many reported making progress in a treatment program only to be discharged without sufficient support post-treatment, leaving them vulnerable to return to use. Often recovery happens over years, and people may fall in and out of different stages of recovery throughout that time.

Why did I- why did I relapse? I don't know. I don't know. I don't know. I really don't know I- I use the excuse of boredom. I use the excuse staying clean is hard. It's… I like being quote unquote ‘effed up during the day, so I can get through the day… because my head is- being inside my head sober is kind of a… kinda a trip. My head is kind of screwed up. I have friends that they're so thankful that they got arrested and they had to spend a few months in jail, you know, and then there's people that were grateful for those 30 days that they got to go to treatment. You know, some people, um, just waking up in an ICU was enough, and then others, like a friend passed away. I mean, there's so many different roads to recovery. I think it's important to understand they all have an important role, you know, and just understanding that like, everybody kind of finds their way, but all of the options have to be available.

Male, 30s, used opioids in the past

It's not so much the drug, the drug is the drug, but then there's the lifestyle then there's the wreckage that it causes emotionally and mentally and financially, physically, you know, all these things that- that now are going unacknowledged in terms of the support, and the- the resources needed. And so people think, oh, I can, they just need to get off the drug and everything's going to be fine. And usually when we get off the drug, we're worse than when we started out. So it's really hard. It's really, really hard. I hated recovery. It was saving me, but I hated it because it just didn't feel good. There was still all this emotional pain and hurt and an agony, you know, there was just, there's just this overwhelming sense of defeat in recovery. And I just, just like, if this is what it is, what the hell is the point, and so I started to associate with people again, who- who were using and when I had first gotten clean... When you start using heroin, it numbs you from everything, the good, the bad, the indifferent, the significant, insignificant. It just… it robs you of everything. So when you, when you're trying to come out on the other side, you're so exposed and you're so raw and you're so overstimulated in so many areas and it's hard. It's really hard to kind of get a grasp on that…. You know, it's a lot easier to ride a bike downhill than it is uphill and, you know, being able to stick with changes when it's painful, and then be shamed by people when- when- when you don't. There's just a lot of pain. There's just so much pain associated with it.

Female, 30s, used opioids in the past

Furthermore, it was also common for people to change lanes on their recovery path. Several participants mentioned how someone must be ready to make a significant change in their life, and that readiness may arrive at different times and for different reasons for each individual.

I guess it's like I said, like basically, it's just like a switch flipped in their brain like they just started caring about their family all of a sudden. Like they wanted to be around their kids and they almost died or fucking you know, even though they've almost died six times before, this time was the one that fuckin’ woke them up, like I don't know, like it's honestly a mystery to me. They got so tired of the drug that they just, they don't want to do it anymore. That, that's actually probably a big one, like you get so tired of doing this shit.

Male, 20s, currently using opioids

There's so many reasons, a lot of reasons they just want to get out of the lifestyle, the life, the lifestyle is exhausting, they have a family, or they got kids or they see that other people in recovery themselves and how good they're doing and that motivates them to do that themselves.

Female, 30s, used opioids in the past

There's no sort of magic point, everybody's different. And so you really have to kind of meet people where they are in that process. You can't force them. No amount of rehab is going to force them, no amount of, you know, sort of medication or anything. They just have to kind of get to that point. And that it's really important to kind of let people know that they're supported and that they can do it when they're ready. … it's not a linear process. You know, it's kind of a complicated process.

Female, 20s, used opioids in the past

You know, it also depends on where the other person is though. It's not going to be as effective if someone's not ready. Like I can help people all day long, you know, and they can tell me what I want to hear, or what they think I want to hear. And then go out to… right away and use.

Male, 30s, used opioids in the past

Only if you’re ready… You’re not going to be forced into it, if you get forced into something then you’re doing for them. Once that person or that reason is gone, guess what? You're going to still have that monkey on your back and you’re going to be screwed again. You’re even more desperate at this point because you, you're now- you're out of that circle that you were in… you're in a different circle and you're going to be more desperate to have that you're going to risk it and do more harm.

Male, 30s, currently using opioids

In summary, there are multiple push factors that shape one’s individual motivation to initiate the recovery process. Since motivating factors differ from one individual to another, it is difficult to predict when an individual will be ready to consider treatment or recovery and what will precipitate a change in behavior.

### There are significant logistical and psychosocial barriers to services, treatment, and recovery that do not account for the complexity of opioid misuse

The participants spoke about a myriad of barriers to accessing and participating in services and treatment, and to initiating or maintaining recovery. The barriers were often systemic and included logistical obstacles, such as limited and siloed facilities with restricted hours of operation. They also included psychosocial obstacles like stigmatizing, judgmental, and dogmatic services that, instead of facilitating recovery, resulted in alienating and ostracizing those in need at their most vulnerable state.

#### Logistical barriers

Everyone agreed that there were basic needs barriers to accessing services or initiating/staying in recovery that included lack of housing options, transportation, food security, and financial difficulties. There was also a broad perception that Medicaid did not cover many important services. Everyone reported that harm reduction and medications for opioid use disorder (MOUD) are vital, but many said that these services had limited capacity and were under-resourced, which led to fewer hours of operation and confusion about the location and availability of services.

It's ridiculous right now. They [harm reduction services] aren’t even open right on site. They they're all over town and most of the time I can't get across… across town, to get a needle exchange.

Trans-woman, 40s, currently using opioids

Well, you know, I just got the minimum once a month counseling [at the methadone clinic], but at the time, yeah. I didn't know. I should be getting more counseling. So my tribe pays, I now know this, that my tribe pays for counseling or they do counseling, but they also pay for medication. I didn't know that at the time. And I was paying out of my own pocket, but then when I decided to get on Suboxone, Suboxone is expensive. So, I even had trouble at my own clinic getting help for my addiction. I went in, I went to the doctor, I asked them if I can get this filled and the doctor's like, we don't fill that here. And he gave it back to me. And the only time that- the only reason why I was able to get help was because I started crying. But also, because I had an auntie that worked in the clinic that she was able to help me get it paid for. But if I didn't know somebody, and if I didn't really cry, then he would've just let me go. He would have just said, like, you know, we can't do anything for you.

Female, 20s, formerly used opioids

Everything is so hard with all the, I don't know if it's government or like the restrictions or grants or whatever it is, but they make everything so hard. And when you have someone who it's hard for them to get out of bed and brush their teeth and you want them to go and jump through all these hoops… we need to simplify it more and just make it not as hard, not as hard.

Female, 30s, formerly used opioids

Some participants reported that treatment centers were, at times or in certain circumstances, helpful. However, some viewed these services as predatory, only concerned with making a profit, and selling a flawed product not based on evidence. Many treatment centers had differing requirements that made entering or staying difficult (i.e., sober on entry, no smoking, no couples, no sugar, etc.). Additionally, the lack of community-based recovery support and resources upon discharge increased susceptibility to return to use, risky practices, and overdose. Most services were not perceived as taking a holistic approach to the complexity of substance use disorders. Many respondents said that they felt programs and services functioned separately from each other without collaboration, so did not take advantage of aspects of other services that could compliment what each program may be lacking.

I should have recognized that I was that sick. And rehab, you know, it's… it's a business, you know they're there to make money, and that was also very clear, you know.

Female, 40s, currently using opioids

You know, some people are in it, because they want to… they want to help people, but then other people are just there for the paycheck and you can tell the difference between the two people and a lot of people will look down on you for what you do and it sucks but I mean what can you really do about it, you know.

Female, 20s, formerly used opioids

Like it's always gone when I'm sick and I really, really need help. That's why I'm so desperate and I'm calling them and I ended up having to come back on like a, I don't remember if it was a Tuesday or Thursday and I'm over here. Like I'm on my second day of being sick. So I, there's been a few times where I go there and it's like, I have to be turned away… So it's like, when they turn me away and tell me, I have to come back the next day to sign up or wait two days. I'm like, are you kidding me? So it's, it sucks. I've had to go through that a couple of times.

Female, 30s, formerly used opioids

#### The justice system

The theme from responses was that the justice system, law enforcement, and current drug laws seemed to contribute to the cycle of substance use and drug-related crimes. The justice system offered little, or no, services during incarceration and drug courts were reported as mostly unsuccessful long-term and in need of significant revision or replacement by better evidence-based services. The justice system was also reported as a major source of stigma and posed additional barriers (post-release) to accessing housing and other basic needs. Many respondents indicated that justice-involvement was ineffective to behavior change. Many others cited the “drug war” as a primary driver of barriers to reentering society, engaging in risky behavior, overdose deaths, and fear.

January 2014, I got locked up and I they're, they're going to send me to prison and, and, and I, I was happy to go to prison. That's like part of the cure, people are like, you know, you're going to go to prison and that should make you shy away from doing drugs and a criminal lifestyle. It doesn't work like that. We know that we're probably going to go to prison.

Male, 40s, formerly used opioids

They're killing people and they don't even know it. By putting people in jail for this stupid shit and then letting them back out they are killing them.

Male, 40s, currently using opioids

And then eventually we got pulled over, and they throw us in jail. I got like internal possession. And I took like, so I took the drug court program and they let us all out, and then I relapsed a few times while I was waiting on drug court. Until I was ‘fine like I need to do something’ and then that whole… I tried getting on Suboxone like just down here. Then [harm reduction worker] showed up and helped me do all that. So I'd been like white knuckling it for about a month before I finally talked to (harm reduction worker) and got on Suboxone. And so I just, I had to try some, you know, I had to do something different.

Male, 30s, formerly used opioids

I was in the psych ward for a couple of weeks. I lost it there. I was going through like some extreme psychosis. I wound up catching a charge while on the psych ward. Um, I got arrested, I got a disorderly conduct and I just remember being handcuffed and then taken to the federal jail, thinking to myself that like, I went there to go- go get help. And now I have another charge. And like, just thinking how hopeless it all was.

Male, 30s, formerly used opioids

Well, I mean, [harm reduction organization] is actually pretty cool. They're all about harm reduction, you know, which is really I mean, that's the best...dude there is no winning a war on drugs. There is no winning that fucking war. No matter what, somebody fucking… somebody's going to lose in this fucking shit. The populace isn't gonna win. The government isn't gonna win. Indeed, you know. And so the best that we can hope for is harm reduction. You know, like the Narcan being fucking out there when people need it.

Female, 30s, currently using opioids

These are fucking failed policies across the board here in the United States. State policies, federal policies across the board have failed. We can see from the 80s that they failed, we can see from the 90s that they failed, we can see from the fucking early 2000s that they, we can see from his last decade, they fucking failed dude. So how long you guys can keep doing this shit. You know?

Female, 30s, currently using opioids

#### Psychosocial barriers

Stigma was reported as a significant barrier to accessing any type of services and kept people in recovery from maintaining recovery. Isolation and lack of hope was also a significant barrier to remaining in recovery, and stigma only reinforced these feelings. Participants in particular spoke about stigma from medical personnel and law enforcement.

I've been treated like dirt so many times in the hospital that I've literally not went in for care whenever I've needed it, because I knew that they would be mean to me because I was on methadone or because I had track marks or, you know, all young, like you know, and I've had them being mean to me like because, because I had track marks, and I was raped…. After they found my track marks it was like the whole atmosphere changed. I must have been turning a trick and it must of went wrong, and they acted like I deserved to be raped or something, you know like, or that I was drug seeking.

Female, 30s, formerly used opioids

I remember I was withdrawing really bad and I went into the ER and I wanted to check myself in and they just let me sit there for hours and I wound up just freaking out and just pulling my IV out and leaving.

Male, 30s, currently using opioids

I don't know the officer's name, but I'll never forget. He pulled me out back and very brutally was very blunt with me. And in the sense of, he had compassion in his eyes, but he was like, what the fuck are you doing with your life? He said, what- what are you doing? You're pregnant. You're about to bring a baby into this world. And you're here doing this. Like, what are you doing? And I didn't have an answer.

Female, 30s, formerly used opioids

Some reported that Alcoholics Anonymous, Narcotics Anonymous, and other 12-step programs could be useful, but many said they also tend to be inflexible and therefore alienate some people. There was a described need for support groups that are more holistic and accepting of people wherever they are in the process towards recovery. Dogmatic or restrictive services and/or inflexible close contacts (services or family that cut PWUD out for using drugs or relapsing) mostly served as alienating to the participants.

They're almost like a cult in some ways, because if you're not one of them, they treat you like shit. You know, like they really do. And so, if you don't have the story that you're supposed to have or if you're- you're taking Suboxone they look down on you. I didn't do real well with that…

Male, 30s, currently using opioids

Yeah, I was clean. I'd already went through the withdrawals and detox. It was mainly getting used to all the rules. And all the rules in rehab are something else for people who are coming off the street, are coming out of jail and aren't used to having any rules. I almost left on my first day and it was because all these rules… they were barking rules at me and like watching and waiting for me to mess up… It was very stressful for me.

Female, 30s, formerly used opioids

I got a lot of negative backlash from the- the 12 step supporters because I decreased my attendance of meetings. So when I would go to a meeting, it wasn't with open arms. It was, ‘I needed to call my sponsor’ and ‘I needed to start working a program’. And who do I think I am not coming to meetings? Do I think I'm better than, you know, am I different? It was just, it was very, it was very harsh, and it was very, uh, I just ended up saying, fuck you and stopped going to meetings entirely. So then I was just down to one recovery service support thing, and that was my job. So it really narrowed down my choices.

Female, 30s, used opioids in the past

Participants spoke openly about the challenges faced when seeking help, including barriers to accessing harm reduction, treatment, and recovery services. Additionally, they often reported difficulty navigating the justice system, service intake and limited hours, and strict rules of different programs.

### Compassion, hope, and a sense of purpose are crucial facilitators to recovery

Connecting to purpose and meaning was strongly linked by participants to maintaining recovery and, in many cases, necessary to initiating recovery. Participants gave examples of times they felt valued, which they said provided hope and helped to heal internalized stigma they had absorbed from feeling dehumanized by providers, law enforcement, community members, and family members. They reported the great importance to their recovery of having hope, feeling that others tolerated them, not feeling that they were given up on, and how important it was to them that people in their lives were willing to meet them where they were in the process. The participants also said a sense of belonging was crucial to maintaining recovery. Harm reduction services helped people by keeping them safe, offering a safe and often non-judgmental space, facilitating referrals, and providing other resources. Peer support was seen as an incredible resource for PWUD and those in recovery and was perceived as more effective than counselors or social workers without lived experience.

I mean, my, even just my- my lived experiences kinda give me, I don't want to say an understanding of somebody's situation, but it's, so it gives me empathy and compassion, um, about it. And just a little bit of understanding of the struggle that people can experience. I know I would not be where I'm at right now, if I wasn't given a chance and I- I believe heavily that every person deserves a chance. And, you know, like I said, a little while ago, purpose is a powerful thing. And if like the people that come and they do have their experiences and they've struggled, you know, and something as simple as a job, or they can use some of their experiences to help other people like lights this purpose, and I've seen people thrive and to be able to share my experiences with others.

Male, 30s, formerly used opioids

I guess it's just all about changing my circle of friends, you know. Finding people who are in recovery as well, instead of sitting there and trying to hold onto friendships that I know that aren't good.

Female, 20s, formerly used opioids

The participants articulated the importance of compassion, connection, and not having others give up on them. One question we asked the participants is what they would want others to know about PWUD. Over and over, they said they wished for tolerance, understanding, and to be seen as real people.

It's not just the people that, like, are on the streets or anything. You know, it's a son, daughter, it's the doctor. It's the lawyer it's, you know, like it's anybody. And I think it's really important to know that when we stop labeling a specific group of people as the reason for this, and see that it's something that affects everybody, then you can come to the table more as a community and try to solve it. Because so many people sit there and are like, ‘oh, that's not my problem’. But what they don't know is, it’s probably their wife or kid, or it is their problem. They just don't know it yet.

Male, 30s, formerly used opioids

I mean it always goes back to just riding a bicycle. When you put a little child on a bicycle and they go peddling off and they fall down, if you just let them fall down and you're like ‘Oh, he never gonna ride a bike’ like you do not do that. No, you continue to train him to where he learns to ride a bicycle. You understand it is not his fault, you got to understand where he is at, not where you want him to be. And you know if we did that, with people… to have the experience for me, to be like hey, you know I kind of- I kind of read through your–and excuse my French–but your bullshit… if you want to keep selling me that go ahead. But you know, I am not going to give up on you.

Male, 34, formerly used opioids

Many participants recalled memories that profoundly altered their sense of belonging or purpose. Others discussed the importance of having someone to connect to who had similar experiences and a non-judgmental, compassionate regard for PWUDs. Moreover, others discussed the importance of having individuals not give up on them, wherever they were in the continuum of drug use.

## Discussion

The 35 participants we interviewed gave us unique in-depth information about their experience with opioid use, accessing services, and the recovery process. Our findings indicate that in almost every narrative, there was some sort of traumatic situation(s) or experience(s) that influenced their proclivity for drug misuse, and that opioids came into their life as a method to cope with painful events. Others described growing up with family members who had a history with substances, which often precipitated their own use. Despite the temporary relief that opioids offered, participants described the ongoing cycle of physiological dependence that perpetuated their opioid use like “*hooks”* or “*being shackled”* and feeling *“trapped.”* They also highlighted how the process of extricating themselves from opioid misuse was most often complicated and entropic, and varied for each individual. Even though almost all the participants currently misusing opioids expressed a desire to break their dependence, they reported many barriers to accessing and participating in services. The barriers were logistical, like too few services with difficult hours or exclusionary rules, or psychosocial, like encountering stigma and social isolation from their support system and from within the services themselves. The participants’ challenges encountering these barriers underscored the need for compassion from the community and an internal sense of purpose to help aid their recovery in the longer term.

The participants shed light on the role opioids play in alleviating emotional pain—people used opioids because the drugs served a specific function. Their responses corroborated the literature on the role of trauma as a facilitator of substance dependence; Ponnet et al. suggest that drug use is a rational decision to achieve a specific outcome [24]. When considering people’s options for relief from the fallout of adverse childhood experiences or traumatic experiences, self-medication is a rational decision as an avoidant coping mechanism [25–29]. People used these substances because the opioids made them feel good, or *“like being next to God,”* or feel nothing at all, and were *“consistent”* in what they experienced as an unpredictable and tumultuous reality. Yet the narratives also describe how opioids can pull people towards chaotic or problematic use, which further exacerbates prior trauma and emotional stress. Services that address the underlying risks to substance use were found to be helpful to one participant who described the arduous but ultimately effective process of confronting past trauma that, until dealing with it, had been perpetuating her attempts to numb the pain. Trauma-informed care was not often discussed as part of the services our participants had experienced, so services that haven’t incorporated this may consider revisions to their strategy to help improve longer term outcomes [19]. It is important to note, however, that in order to properly address prior trauma, an individual’s basic needs must be met; services should also first help safeguard basic physical and emotional needs for their clients.

Participants described motivation for entering treatment or recovery as personal, individualized, and messy. The process of change was discussed as *“not a linear process”* rather *“kind of a complicated process”* similar to what Prochaska et al. described in their work on the transtheoretical model of change [30]. These perspectives were also supported with findings from Downey et al. that intrinsic motivation is a leading factor in initiating and maintaining recovery, more than other legal or health factors [31]. In fact, one of the least cited reasons for people to cease use is participating in formal treatment [32]. Additionally, stigma and criminalization of drug use in the US have contributed to constructing societal attitudes of moral judgment against PWUDs [33]. Participants discussed their experiences of social separation or rejection by family members and other supports in their networks. This experience of disconnection, isolation, and the internalized stigma further propagates a damaging cycle and is often a barrier to seeking services or supports [34]. Earnshaw asserts that behavioral health providers must be part of changing structural stigma by adopting stigma-free language and challenging policies that criminalize people who use substances [35]. Reforms to the criminal justice system in Nevada and elsewhere could benefit by considering implications of decriminalizing drug use, and by incorporating more aid to risk reduction, peer recovery support services, on-demand access to MOUD, and for related social determinants of health (e.g., aid with housing, transportation, food, clothing, etc.) [36].

This current wave of the opioid epidemic is inherently dangerous. Prescription medications with known dosage and ingredients has shifted to the opaque illicit market in which the supply is largely adulterated by illicitly manufactured fentanyl and other unsafe analogues. Participants described the tension between centering their daily activities around acquiring and consuming opioids and an acute awareness of the dangers of the illicit market. In Canada, there are services that to address these dangers by including drug safety checking, safe consumption sites, access to naloxone and MOUD as well as a movement toward safe supply interventions [37]. Ivins et al. suggest these harm reduction strategies as effective public health programs that lower risk of using adulterated products and subsequently, fatal overdoses. Moreover, providing alternatives to illicit drugs provides individuals the time and opportunity to focus on their health and wellbeing [37]. Participants who had sought harm reduction and treatment services described various barriers to access including insurance, paying for treatment, trying to manage changing locations and hours, and convoluted intake processes. Services meant to help were sometimes harmful, as people experienced negative provider attitudes and expressed frustration of treatment programs being a *“business, you know they’re there to make money.”* We suggest reforms to Medicaid in Nevada that enhance or clarify policies for people to access treatment and medication more easily. Furthermore, access to MOUD is limited to intake at opioid treatment programs. The intake process and hours of operation at services should be adjusted, refined, and expanded to streamline safe and effective aid to those seeking treatment [38]. We suggest that treatment programs work diligently to advance multiple pathways to recovery and include ongoing post-recovery support or linkages to support. Considering the importance of tolerance, compassion, and hope for our participants in their recovery process, we also propose that along with reducing stigma should be the prioritization of more holistic and compassionate care for those struggling with opioid misuse. McKay argues for services that expand beyond the narrow target of eliminating substance use to investing in programs that make recovery rewarding to clients; that is, instead of focusing only on taking away the harmful behavior, combine that focus with an increase in enjoyable substance-free activities [32]. In addition to addressing underlying trauma and drug dependence, services could also aim to make recovery rewarding in the short-term by developing more peer support, integrate peer recovery support specialists, and incorporate activities that explicitly foster positive relationships, build community, and promote a sense of value and purpose in people’s lives.

## Limitations

Due to time constraints, this study was limited in the number of rural and minority participants that we were able to interview. There was a chance of bias based on our sampling strategy since we worked with specific services for recruitment. We also had a relatively small sample size so our results may not be generalizable to other populations or locations. More investigation is needed to be able to determine more in-depth whether there are significant differences in barriers and facilitators to services in rural areas and for minorities, including Black, Indigenous, and People of Color (BIPOC) and lesbian, gay, bisexual, transgender, queer, (questioning), intersex, asexual, and (agender) (LGBTQIA +) individuals. It would also be worthwhile to investigate the ways in which intersectional stigma, barriers to services, and criminalization are experienced by BIPOC and LGBTQIA + individuals. Additional research could also focus on perceptions of law enforcement, healthcare personnel, and service providers towards PWUDs to determine whether the implementation of trainings or other activities could effectively reduce stigma and implicit bias and improve outcomes. However, our study did have considerable strengths, one being that we did get a cross section of participants that were currently using and those that had used in the past, and a mix of those with housing and those living in encampments. Working with the partner organizations aided the strength of our study, as they provided access to participants with whom they had built trust that were willing to be candid and honest.

## Conclusions

The participants in this study portrayed opioid misuse as a rational choice to escape the emotional ramifications of trauma. However, due to the physiological dependence of opioids, drug policies that criminalize addiction, societal stigma that portrays PWUDs as social lepers, and the barriers to timely access of harm reduction, treatment, and recovery services, PWUDs become trapped in a distressing cycle. Yet the study participants found access to prevention and services for opioid misuse complicated and challenging. They indicated that hope, value, belonging, and purpose are powerful factors in cultivating intrinsic motivation for making positive changes in their life and fostering resilience in the recovery process. Creating more holistic and accessible approaches to drug management, treatment, and inclusive recovery spaces, while eliminating stigma and elevating compassion, could aid PWUD in connecting with supports and reduce overdose fatalities and drug-related harms.

## Supplementary Information

**Additional file 1: **Semi-structured interview guide.

## Data Availability

The datasets used and/or analyzed during the current study are available from the corresponding author on reasonable request.

## Abbreviations

CDC: Centers for Disease Control and Prevention
NMOU: Nonmedical opioid use
BIPOC: Black, Indigenous, and People of Color
LGBTQIA +: Lesbian, gay, bisexual, transgender, queer, (questioning), intersex, asexual, and (agender)
MOUD: Medications for opioid use disorder
PWUD: People who use drugs
