# Supplementary material for: “I'm putting a Band-Aid on a bullet hole the only way I know how:” a qualitative study of barriers and facilitators to opioid misuse and recovery in Nevada
Source: Subst Abuse Treat Prev Policy. 2022 Nov 24;17:76. doi: 10.1186/s13011-022-00503-0 (PMC9694560; doi:10.1186/s13011-022-00503-0)
Supplement: Supplementary file 1 — Additional file 1: Semi-structured interview guide. [file 13011_2022_503_MOESM1_ESM.docx]

Appendix 1: Semi-structured interview guide

**Interview Date: _______________________________**

**Interview Location (participant’s location if conducted remotely): _______________________**

**Participant’s age: _______________________________**

**Participant’s self-identified gender: _______________________________**

**Participant’s housing status: _______________________________**

**Participant’s race: _______________________________**

**Hispanic/Latinx: Y/N**

**Facilitator’s name_______________________________**

*Thank you so much for talking with us today. My name is _______ and I’m working with [Partner Organization] to use the information and insight you provide us in order to make recommendations for how to decrease barriers for accessing services for opioid use. We are going to ask you questions about your life before you started taking opioids, the reasons why you began to take them, whether you’re currently using, whether you’re in treatment or recovery, and what experience you have had with accessing services for opioid use. Speaking with us today is totally voluntary and you do not have to answer any question you don’t want to. We just ask you to be as honest as possible, there are no right or wrong answers. We hope that your perspective will help us to design better access to services that meet people’s needs. Do you still want to participate?*

**Section I: Participant’s history, how they started taking opioids.**

*First, I am going to start out asking you a little bit about how you started to take opioids. I’m interested in finding out a bit about your background because your perspective is really important.*

- Can you start out by describing a typical day for me?
- What were your social support/networks like growing up? Have those changed over time? If so, how?
- Can you tell me about how your first started taking drugs? When did your drug use start? What drug(s) was it [that you started with]?
- Can you tell me a little about your life before you started taking drugs? [Probe: if appropriate, ask about how they were treated by parents, relatives, etc. Ask about what school was like, where they are from etc]
- Do you think those supports had any effect on whether you started taking drugs/opioids? If so, how? Did your social supports/networks change after you started taking drugs?
- When did opioids first enter your life – what type of opioid(s) and how did you first start to take it/them?
- Have opioids affected your life economically? If so, in what way?
- Did taking opioids change your relationship with people, like your family and other social support systems? With whom? How?
- Was there a moment or a time when you started feeling like it interfered with your life on a day-to-day basis?
- Did your sense of self change when you started taking opioids regularly? If so, how?
- What do you see for the future? What role do think opioids will have in your future?

**Section II. Participant’s current opioid use**

*Now I’m going to ask about your current situation with opioids.*

- Can you tell me what function drugs/opioids have in your life, right now? Has that function changed over time? If so, in what way(s)?
- What kinds of drugs did you/do you take? Has this changed over time? If so in what way? Where do you usually get your drugs from? Has that source changed over time, if so how?
- How do you usually take these drugs (injection, smoking, ingesting etc)? [If they inject] Where do you usually get your needles from?

**Section III. Participant’s current employment situation**

*I’d like to ask you about your current employment status. Are you working right now*? [**If yes, go to section III.A, if no go to section III.B**]

**Section III.A:**

- What kind of work do you currently do? How do you feel about the work you’re doing?
- Do opioids play a function in your work life? If so, in what way?
- Can you describe a typical working day?
- Are there obstacles you face in order to perform your job? Is there anything that would help you perform better at your job?
- Does your employer provide insurance?

**Section III.B:**

- Is there a reason why you don’t currently have a job? How do you feel about it? [Probe to see if they want a job, and if so, what they think would help facilitate getting a job]
- [If they are currently using]

**Section IV: Experience in treatment or recovery**

*Now I’d like to talk to you about your experience with accessing services. First, are you currently using opioids or other drugs? Are you currently in treatment (whether you’re also currently using drugs or not)? Or are you out of treatment and consider yourself in recovery?* **[If currently using opioids/drugs go to section IV.A, if currently in treatment, go to section IV.B, if currently in recovery and out of treatment go to section IV.C]**

**Section IV.A: Currently using**

- How would you describe your top five needs right now? (If they mention stopping use/getting treatment—ask what successful treatment would look like for them. If they do not mention quitting/treatment, say, “I noticed quitting/getting into treatment wasn’t on your list. Can you tell me more about that?”)
- Have you ever looked into any services for drug use? If so, were there any services that you have found helpful? Are there services that did not help? (Why?)
- Have you ever been in treatment for opioid use?
  - **If not**: What are your feelings about your current drug use? Have you ever thought about cutting down or quitting? If so, have you considered or participated in any services that may help you with it? **If so:** what types of services? Tell me about your experience(s). Are there ways that made it difficult for you participate in these services? Are there ways that would motivate you or make it easier for you to participate in these services? (Probe to see whether stigma, social/cultural norms, religion, waitlists/intake/financial barriers had an effect)
  - **If so**: What was your experience(s) like? Why did you leave? Would you consider going back to these services? Why/why not? Are there ways that would make it easier for you to enter and stay in these services? How do you think the services you were in could improve? Was there any aftercare or follow up services?
  - Did you ever take buprenorphine or methadone for treatment? If so, can you describe the circumstances of taking it? [Probe whether given as part of a program or on the streets]
- Do you have friends who have participated in any services? Did they tell you about their experiences at all – what were your impressions from what they told you?
- Have you or anyone you know experienced an overdose? Can you tell me about that/those experience(s)?
- If you were with someone who overdosed, what would you do? Have you ever heard of the 911 Good Samaritan Law? Where did you hear about it? If so, what do you think about it?
- Have you or anyone you were with ever used naloxone/Narcan (an opioid overdose reversal drug)? If so, do you know where you/they got it? Are you aware of places that distribute Narcan?

**Section IV.B: currently in treatment**

- You mentioned you’re currently in treatment. Can you describe for me what you think your top five needs are right now?
- Is this the first time you’ve entered treatment? If no, can you tell me about the past time(s) you were in treatment? Why did you leave? What brought you back? Were there any factors that motivated you or made it easier for you to get treatment? (Probe to see whether stigma, social/cultural norms, religion, waitlists/intake/financial barriers had an effect)
- Can you tell me about what kind of treatment it is? How did you find out about it (referred, found themselves)?
- How long were you taking drugs before you got into treatment? Can you tell me about your experience entering into treatment - how easy or difficult was it for you to get in? What was the intake experience like? [If they had to wait] Why did you have to wait, what was that experience like?
- When/where did you learn about this treatment?
- What do you think about the treatment you’re currently in – can you tell me the things you like about it and the things you don’t like about it?
- Are there ways that made it difficult for you to enter treatment and stay in treatment?
- Are there ways that would make it easier for you to enter treatment and stay in treatment?
- How do you think the treatment you’re in could improve?
- Did you/have you taken buprenorphine or methadone for treatment? If so, can you describe the circumstances of taking it? [Probe whether given as part of a program or on the streets]
- Do you have friends who have participated in any services? Did they tell you about their experiences at all – what were your impressions from what they told you?
- Have you or anyone you know experienced an overdose? Can you tell me about that/those experience(s)?
- If you were with someone who overdosed, what would you do? Have you ever heard of the 911 Good Samaritan Law? Where did you hear about it? If so, what do you think about it?
- Have you or anyone you were with ever used naloxone/Narcan (an opioid overdose reversal drug)? If so, do you know where you/they got it? Are you aware of places that distribute Narcan?

**Section IV.C: currently in recovery**

- You mentioned that you are currently in recovery and out of treatment. Can you describe for me what you consider your top five needs are right now?
- Did you ever seek out formal treatment? Did you enter into a treatment program more than once?
- Can you tell me about what kind of treatment you were in? How did you find out about it (referred, found themselves)?
- Were there services that were helpful in your early recovery? What was most helpful to sustain recovery?
- Was there a factor (or factors) that pushed you into initiating recovery? Can you tell me more about that experience?
- What would you say are the most helpful things for you sustaining your recovery?
- **IF THEY WERE IN TREATMENT:** How long were you taking drugs before you got into treatment? Can you tell me about your experience entering into treatment - how easy or difficult was it for you to get in? What was the intake experience like? [If they had to wait] Why did you have to wait, what was that experience like?
- What did you think about the treatment – can you tell me the things you liked about it and the things you didn’t like about it?
- Were there ways that made it difficult for you to enter treatment and stay in treatment?
- Were there ways that made it easier for you to enter treatment and stay in treatment?
- Do you think there are ways that the treatment you were in could be improved?
- Did you/have you taken buprenorphine or methadone for treatment? If so, can you describe the circumstances of taking it? [Probe whether given as part of a program or on the streets]
- Do you have friends in recovery? Have they told you about their experiences? What is your impression of what factors were most helpful to them in initiating or sustaining recovery?
- Have you or anyone you know experienced an overdose? Can you tell me about that/those experience(s)?
- If you were with someone who overdosed, what would you do? Have you ever heard of the 911 Good Samaritan Law? Where did you hear about it? If so, what do you think about it?
- Have you or anyone you were with ever used naloxone/Narcan (an opioid overdose reversal drug)? If so, do you know where you/they got it? Are you aware of places that distribute Narcan?

**Section V: Messaging for treatment**

- Have you heard or seen any messaging about getting treatment? Where did you see it? What did you think?
- What other messages that encourage treatment do you think should there be? Where should they be put, how are the best ways to reach you? What kind of messaging would most motivate you? Who is a trusted source of information for you? Where do you look first for information?
- What do you think influences the people around you to get treatment, what do you think their trusted sources of information are?
